# Preparation and Comparison of Properties of Three Phase Change Energy Storage Materials with Hollow Fiber Membrane as the Supporting Carrier

**DOI:** 10.3390/polym11081343

**Published:** 2019-08-13

**Authors:** Li Xiang, Dajun Luo, Jingkui Yang, Xin Sun, Yating Qi, Shuhao Qin

**Affiliations:** 1 College of Materials Science and Metallurgy, Guizhou University, Guiyang 550025, China; 2 National Engineering Research Center for Compounding and Modification of Polymer Materials, Guiyang 550014, China

**Keywords:** energy storage materials, hollow fiber membrane, supporting carrier

## Abstract

Herein, we have used a hollow fiber membrane as a support layer material to encapsulate paraffin in order to prepare a phase change energy storage material. The phase change energy storage materials with three different support layers were successfully prepared and various properties were systematically characterized. There are also few reports on the use of hollow fiber membranes as the support carrier for the preparation of phase change energy storage materials. The significance of this work is in exploring the use of hollow fiber membranes as a support layer to prepare phase change energy storage materials. In addition, the choice of support carriers for phase change energy storage materials was enriched. Both the hollow fiber membrane columnar hollow portion and the pore structure of the membrane wall could be used to encapsulate paraffin, which makes it more advantageous than the other support materials. The SEM characterization confirmed that paraffin was successfully encapsulated on the membrane wall and columnar hollow part of the membranes. The TGA results indicate that the introduction of the membrane as an encapsulation carrier delayed the decomposition of the composite phase change energy storage materials. The activation energy during the decomposition in the three different phase change energy storage materials was calculated by the decomposition kinetics. Among the three fiber membranes explored in this work, the polypropylene (PP) encapsulation membrane performed better than the other two encapsulation membranes in terms of encapsulation content of paraffin, porosity, latent heats, thermal stability, and activation energy.

## 1. Introduction

With the advancement of science and technology, human demand for energy has also greatly increased. However, the low utilization rate of energy has led to wastage of a large amount of energy. Hence, it is essential to develop novel energy storage technologies. Among the several techniques explored to improve the energy utilization rate, Thermal energy storage technology (TES) has attracted increasing attention and has been rapidly developed. There are three types of TES, namely latent heat storage, sensible heat storage, and chemical reaction energy storage. Among these, latent heat storage has been extensively studied due to its high energy storage density, high thermal efficiency, and exothermic mode for constant temperature or near constant temperature process [1]. This energy storage method aids in collecting excess heat when there is more heat than required and releases the stored heat when there is a shortage of heat [2]. This method of storing heat energy is an effective means to maintain the balance between heat supply and demand [3].

Latent heat storage materials are also called the phase change materials (PCM). PCM have a heat storage function which can absorb or release a large amount of energy through phase change and has the advantages of high heat storage density, low cost, and excellent chemical stability [4,5,6]. PCM have played an increasingly important role in the research of green energy and environmentally friendly materials owing to several advantages [7,8,9]. Nowadays, PCM have applications in various fields such as waste heat recovery, building energy conservation, solar energy utilization, and other thermal energy storage systems [10,11,12].

These encapsulation methods of PCM can be divided into two categories: microencapsulated PCM [13,14,15] and PCM in spatial mesoporous structures [16,17]. The first patent for oil-filled microcapsules was applied in 1953. Zhang et al. [18] synthesized microcapsule phase change materials based on n-octadecane core and silica shell via interfacal poly-condensation. Luz et al. [19] carried out microencapsulation of polyrotaxanes (PRS) paraffin by means of suspension-like homo-polymerization of methyl methacrylate (MMA) and polymerization with methyl acrylate (MA) and methacrylic acid (MAA). Py et al. [17] proposed a supported phase change material formulated by impregnating paraffin with a compressed expanded natural graphite matrix using capillary force. Ye et al. [20] prepared a new stable phase change material, namely polyethylene-paraffin compound (PPC) which is a compound consisting of paraffin as a dispersed phase change material and high density polyethylene as a supporting material. 

Among all the studied phase change materials, paraffin is the most promising solid-liquid organic phase change material. Due to its low cost, non-toxicity, low chemical activity, and non-corrosive properties, it has had wide applications [21]. However, paraffin is prone to leakage during the phase change process, which greatly restricts its applications [22]. At present, encapsulation of paraffin in a carrier material to obtain a shape-stable composite phase change material (ss-CPCM) is one of the most promising methods for overcoming liquid paraffin leakage. The shapes of ss-CPCM are usually particles [23,24,25,26,27], foam [28], fibers [29], and other shapes [30,31].

However, the shape of ss-CPCM limits the choice of support material. Various ss-CPCM have been prepared by impregnating PCM into porous supporting materials, such as expanded vermiculite (EVM) [32], expanded perlite [33,34], diatomite [35,36], graphene oxide [37], and expanded graphite [38,39]. There are also few reports on the use of hollow fiber membranes as the support materials for the preparation of phase change energy storage materials. Hollow fiber membranes have the advantages of stable shape and porosity and are commonly used in the field of membrane separation technology [40]. Both the membrane wall and the columnar hollow portion of the hollow fiber membranes can be used to encapsulate paraffin, which results in a hollow fiber membrane that is superior to the other support materials. This property increases the amount of paraffin encapsulation content with a simultaneous improvement in the performance of energy storage.

In this paper, three hollow fiber membranes developed by our research group were used to encapsulate paraffin, and three phase change energy storage materials with different support materials were successfully prepared. The materials for three different hollow fiber membranes were poly (vinylidene fluoride) (PVDF), polypropylene (PP), and polysulfone (PSF). The three phase change energy storage materials after encapsulation of paraffin are referred to as PVDF encapsulation membrane (PVDF EM), PP encapsulation membrane (PP EM), and PSF encapsulation membrane (PSF EM), respectively. As all the three hollow fiber membranes belong to the category of microfiltration membranes, various properties of the three phase change energy storage materials were characterized and compared.

## 2. Experimental

### 2.1. Materials and Equipment

#### 2.1.1. Materials

PVDF (poly vinylidene fluoride, Solvay 6020, *M*_w_ = 6.8 × 10^5^) was supplied by Solvay Solexis, Brussels, Belgium. The diluent mixture is composed of PC (Poly(propylene carbonate), *M*_w_ = 236.694) and DOTP (Dioctyl terephthalate, *M*_w_ = 390.56). 

PP (polypropylene, T30s, *M*_w_ = 8 × 10^4^) was supplied by Lanzhou Petrochemical Co., Ltd. (Lanzhou, China). The diluent mixture is composed of DOP (Dioctyl phthalate, *M*_w_ = 390.56) and DBP (Dibutyl phthalate, *M*_w_ = 278.34). 

PSF (polysulfone, Solvay P-3500, *M*_w_ = 8 × 10^4^) was supplied by Solvay Co., Ltd. (Brussels, Belgium). The diluent mixture is composed of DMAC (*N*,*N*-Dimethylacetamide, *M*_w_ = 87.12) and PEG2000 (Polyethylene glycol 2000, *M*_w_ = 44.05). 

Paraffin was purchased from Shanghai Huayong Paraffin Wax Co., Ltd. (Shanghai, China), and its melting point is 48–50 °C. All diluents and Kerosene were purchased from Sinopharm Chemical Reagent Co., Ltd., (Shanghai, China). Silicone rubber was supplied by Guangdong Hengda New Material Technology Co., Ltd., (Guangzhou, China).

#### 2.1.2. Equipment

All equipment used in the experiment were shown in Table 1:

### 2.2. Preparation of Hollow Fiber Membrane

The three virgin membranes were prepared by our research group. In a vessel at 180 °C, the PP/PVDF raw materials and diluent were stirred and dissolved to form dope. Then, the dope was fed into the twin-screw extruder, extruded from the spinneret at 48 mL/min under the metering pump, and quenched in an anhydrous ethanol bath to form a nascent membrane. The diameters of the outer and inner tube for the spinneret were 4 and 2 mm, respectively. The residual diluent mixture within the nascent membrane was further extracted by ethanol. 

The PSF and diluents were fed into the vessel at 70 °C to form dope. The mixture was then extruded from the spinneret at 48 mL/min under the metering pump and quickly immersed in non-solvent (deionized water) condensation. The diameters of the outer and inner tube for the spinneret were 4 and 2 mm, respectively. The residual diluent mixture within the nascent membrane was further extracted by deionized water.

### 2.3. Preparation of Phase Change Energy Storage Materials

All the three hollow fiber membranes belong to the category of microfiltration membranes. The three hollow fiber membranes were treated at 120 °C for 1 h to prevent the shrinkage. The three virgin membranes are placed in a beaker containing liquid paraffin at 70 °C. Thereafter, the beaker was kept in a reactor with an ultrasonic transmitter. In a constant temperature environment, ultrasonic treatment was performed for 1 h to ensure that the paraffin was absorbed into the pores of the membrane wall. The membrane of encapsulation paraffin was quickly taken out, and the liquid paraffin remaining on the surface of the membrane was removed using a filter paper. Then, the three membranes of encapsulating paraffin were sealed with silicone rubber before being used.

### 2.4. Characterization

#### 2.4.1. Morphology of the Membranes Surfaces

In order to clearly observe the surface morphology of the membrane and verify the effect before and after the encapsulation paraffin. The SEM (Quanta FEG 250, FEI instruments, Hilsboro, OR, USA) was used to assess the surface of the membranes. The surface of the membranes was sputter-coated with gold in order to avoid charge accumulation before observation.

#### 2.4.2. Porosity and Pore Size Distribution

The pore size distribution and pore average size of the virgin membranes were determined by an AutoPore IV 9510 instrument (Micromeritics, Shanghai, China). The porosity was calculated by measuring the difference between the weight of the wet membrane and the dry membrane and the density of the working reagent and the membrane. The weighing method [41] was used to test the porosity of the walls of three virgin membranes. The principle of the weighing method is to fill the pores of the membrane with a suitable working reagent. The working reagent should not the cause the membrane to swell or dissolve, or chemically react with the membrane. Besides, membranes should not have adverse effects on the working reagents, and the smaller is the surface tension ratio of the working reagents, the better is the porosity estimation. By comparison of different reagents, kerosene is a good choice as a working reagent. Equation (1) was used to calculate the porosity of the hollow fiber membranes:

(1) $$\varepsilon =\frac{(w_{1}-w_{2})/\rho_{working\;reagent}}{w_{2}/\rho_{membrane}+(w_{1}-w_{2})/\rho_{working\;reagent}}$$

where *ε* (%) is the porosity. *w*_1_ (g) is the total weight of the membrane itself and the kerosene in the pores of the membrane, while *w*_2_ (g) is the weight of the membrane after drying. *ρ_membrane_* (g/cm³) is the density of the three different membrane raw materials while *ρ_working reagent_* (g/cm³) is the density of kerosene.

#### 2.4.3. Percentage Content of Paraffin

The difference in mass before and after encapsulation can be used to calculate the percentage content of paraffin in the encapsulation membrane. Equation (2) was used to calculate the percentage content of paraffin:

(2) $$\mu =\frac{m_{2}-m_{1}}{m_{2}}\times 100\%$$

where *μ* (%) is the percentage content of paraffin. *m*_1_ (g) is the average mass of virgin membrane, while *m*_2_ (g) is the total mass after paraffin encapsulation.

#### 2.4.4. Chemical Compatibility

For the purpose of exploring the chemical stability of the combination of paraffin and three virgin membranes. Attenuated total reflectance FTIR spectra was utilized to characterize the chemical change on the surfaces of paraffin and three encapsulation membranes in the range of 600–4000 cm^−1^ with a resolution of 4 cm^−1^ (NEXUS570, Nicolet, Madison, WI, USA).

#### 2.4.5. Latent Heats and Cycle Reliability

Differential scanning calorimeter (DSC, Q10, TA, New Castle, DE, USA) was employed to test the latent heats and phase change temperature of samples. All the samples (about 5~8 mg) were heated and cooled in the temperature range of 0 and 100 °C at the heating/cooling rate of 5 °C/min in a nitrogen atmosphere. Samples were quickly heated to 100 °C and held at 100 °C for 5 min to erase thermal history, and then cooled to 0 °C at a cooling rate of 5 °C/min and held at 0 °C for 1 min. Finally, the samples were heated to 100 °C again at a heating rate of 5 °C/min. The crystallization curve and the second melting curve were recorded. 

#### 2.4.6. Thermal Stability and Activation Energy

For the purpose of exploring the thermal stability of paraffin and three encapsulation membranes. The thermogravimetric (TG) analysis of paraffin and three virgin membranes was performed by a TA Q50 instrument (New Castle, DE, USA). The heating process in the range 20−600 °C was run with a rate of 10 °C·min^−1^. Samples of approximately 5–8 mg were weighed, and the experiments were carried out under nitrogen with a gas flow of 60 mL·min^−1^.

The Flynn-Wall-Ozawa method [42] was used to calculate the activation energies of the three encapsulation membranes. In a nitrogen atmosphere, the thermal degradation behavior of the three encapsulation membranes was analyzed by selecting heating rates of 5, 10, 20, and 40 °C/min. The activation energies of three encapsulation membranes were calculated by selecting weight loss rates of 5%, 10%, 15%, 20%, 30%, 40%, and 50%. Equation (3) is as follows:

(3) $$\mathrm{lg}F(\alpha )=\mathrm{lg}\frac{AE}{R}-\mathrm{lg}\beta -2.315-0.4567\frac{E}{RT}$$

where *β* (K/min) is the heating rate, *A* (s^−1^) is the pre-exponential factor, *E* (J/mol) is the activation energy, *R* (8.314 J/mol·K) is the gas constant, T (K) is the absolute temperature, and *α* (%) is the weight loss rate. The slope was found by plotting lg *β* versus 1/T, and the activation energy *E* was calculated from the slope.

## 3. Results and Discussion

### 3.1. Morphology of the Virgin Membranes

**Figure 1:**
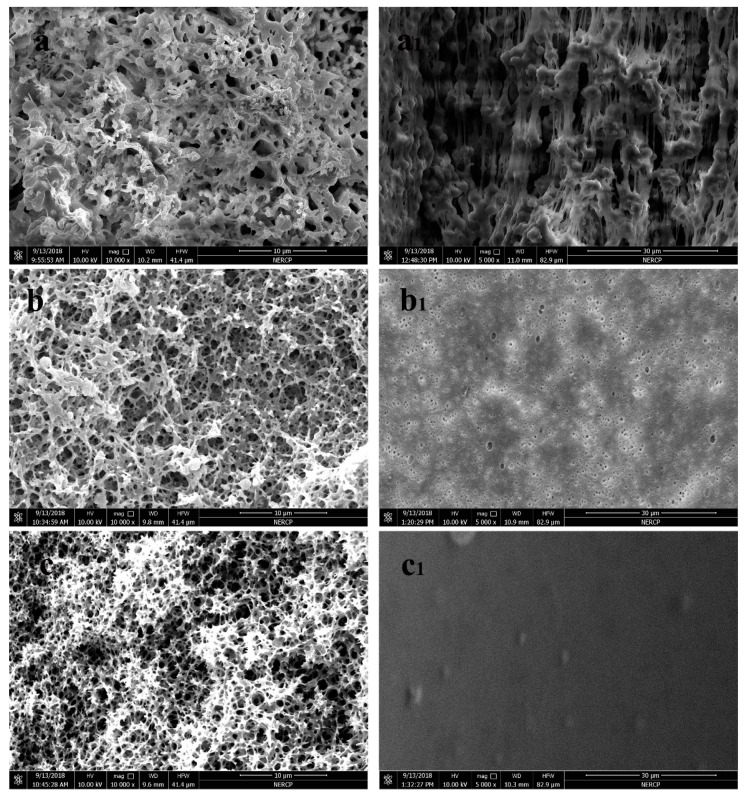
Cross-section and internal surface morphology of three hollow fiber virgin membranes; (**a**) is PVDF membrane; (**b**) is PP membrane; (**c**) is PSF membrane; (**a**), (**b**) and (**c**) are the morphology of cross-section; (**a_1_**), (**b_1_**) and (**c_1_**) are the morphology of internal surface.

The hollow fiber membranes with different raw materials were fabricated to encapsulate paraffin. The performances of the membranes with different raw materials before and after paraffin encapsulation were compared. The matrix used to encapsulate paraffin needed to satisfy the characteristics of being porous. By characterizing the surface morphology, it was found that the surface of the hollow fiber membrane and the membrane walls had numerous pore-like structures, which can be used for the encapsulation of paraffin to prepare composite phase change energy storage materials [43,44]. The morphology of the three hollow fiber membrane sections and the inner surface is shown in Figure 1. As shown in Figure 1, the cross section of the three hollow fiber membranes had a large number of pore structures. The pore structure of the inner surface of PSF in Figure 1 c_1_ was not clear, but the pore structure could be clearly seen in the cross section and inner surface of PP and PVDF. Such a multi-void structure is conducive for the encapsulation of paraffin.

### 3.2. Porosity and Pore Size Distribution of the Virgin Membranes

**Figure 2:**
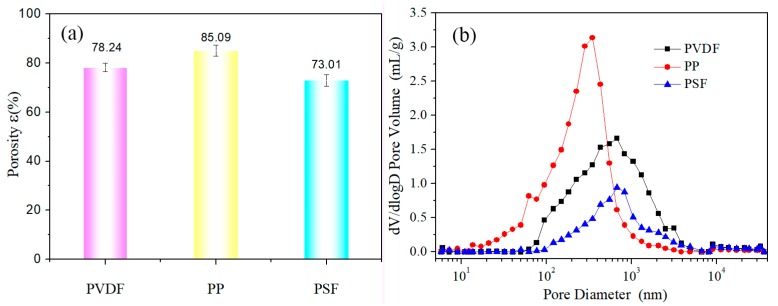
Membrane wall porosity (**a**) and pore size distribution (**b**) of three membranes.

In theory, the larger is the porosity, the higher would be the paraffin encapsulation amount. The quality of membranes before and after kerosene immersion was measured, and the porosity was obtained using Equation (1). The calculation results are shown in Figure 2a. The PP hollow fiber membrane was the best amongst the studied membranes, as the porosity of the membrane wall was the largest, reaching 85.09%. The test results of pore size distribution and average pore size were shown in Figure 2b. The pore average sizes corresponding to the three virgin membranes peaks are 343.5, 256.9 and 360.1 nm, respectively. The height of the peak represents the number of holes, and the width of the peak represents the uniformity of the particle size of the hole. From the width of the peak, the uniformity of particle size of the three virgin membranes are almost the same. The peak height of the PP membrane indicates that the number of pores of the PP virgin membrane is relatively large, and the result also corresponds to the porosity in (a).

### 3.3. Encapsulation Effect of the Membranes

**Figure 3:**
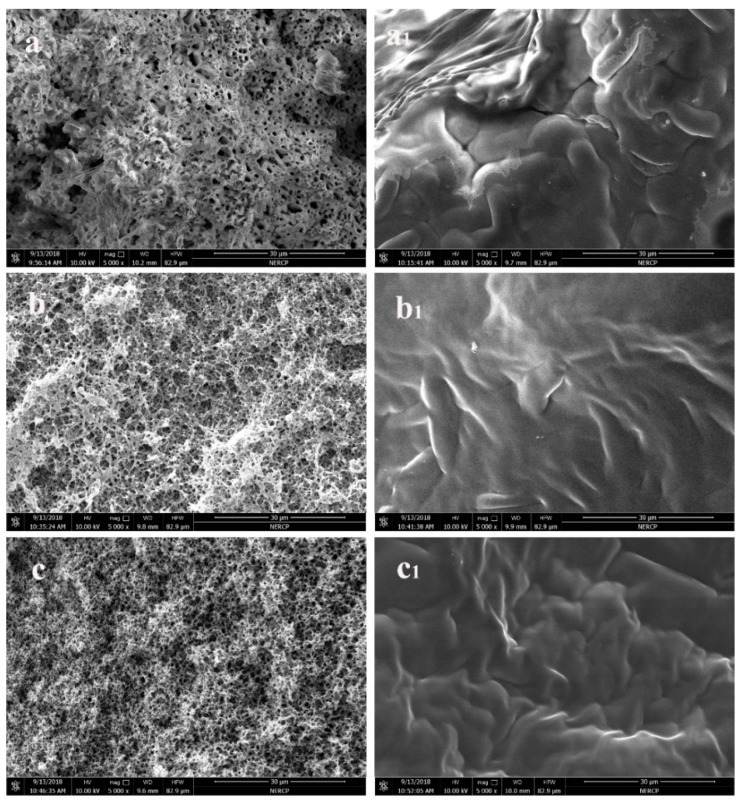
Encapsulation effect of membrane wall Cross-section; (**a**) is PVDF membrane; (**b**) is PP membrane; (**c**) is PSF membrane; (**a**), (**b**), (**c**) are before paraffin encapsulation; (**a_1_**), (**b_1_**), (**c_1_**) are after paraffin encapsulation.

Next, the encapsulation effect of paraffin was investigated. The paraffin encapsulation effect for the before and encapsulation sections is shown in Figure 3; a is PVDF, b is PP, c is PSF, a, b, c are the virgin membranes; and a_1_, b_1_, c_1_ are the encapsulation membranes. As per the Figures of the a, b, and c virgin membranes, small pore structures having a uniform size and a large number appeared in the cross section of the membrane, and these pores were distributed across the membrane wall. These pore structures provide the possibility of paraffin immersing in the membrane wall. As shown in a_1_, b_1_, and c_1_ encapsulation membranes, the pore structure of the cross section almost disappeared. The surface of the pore structure was covered with a layer of paraffin, which implies that the paraffin has entered the pore structure of the membrane wall.

**Figure 4:**
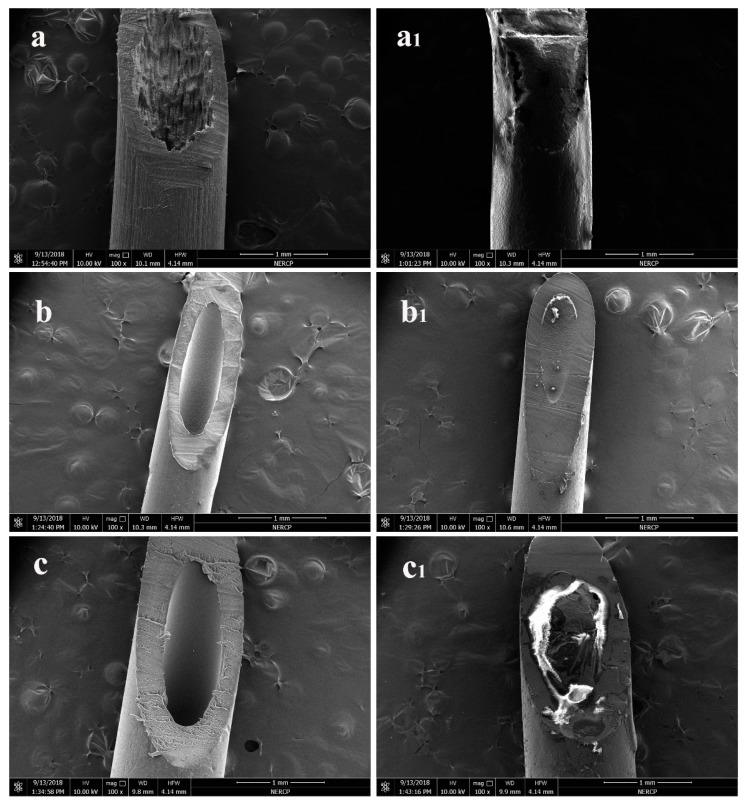
Encapsulation effect of columnar hollow portion; (**a**) is PVDF membrane; (**b**) is PP membrane; (**c**) is PSF membrane; (**a**), (**b**), (**c**) are before paraffin encapsulation; (**a_1_**), (**b_1_**), (**c_1_**) are after paraffin encapsulation.

Figure 4 shows the encapsulation effect of the hollow part. Comparing a and a_1_, and b and b_1_, we found that except for an extremely small number of tiny bubbles, the rest of them were filled with paraffin. Paraffin was filled in the hollow portions of PVDF and PP membranes. The encapsulation effect was ideal. However, on the PSF membrane, the paraffin was not completely filled in the hollow part. This may be due to the dense structure of the inner surface pores of the PSF, which hindered the entry of paraffin and hence, the encapsulation effect was relatively poor. 

### 3.4. Encapsulation Content of Paraffin

**Figure 5:**
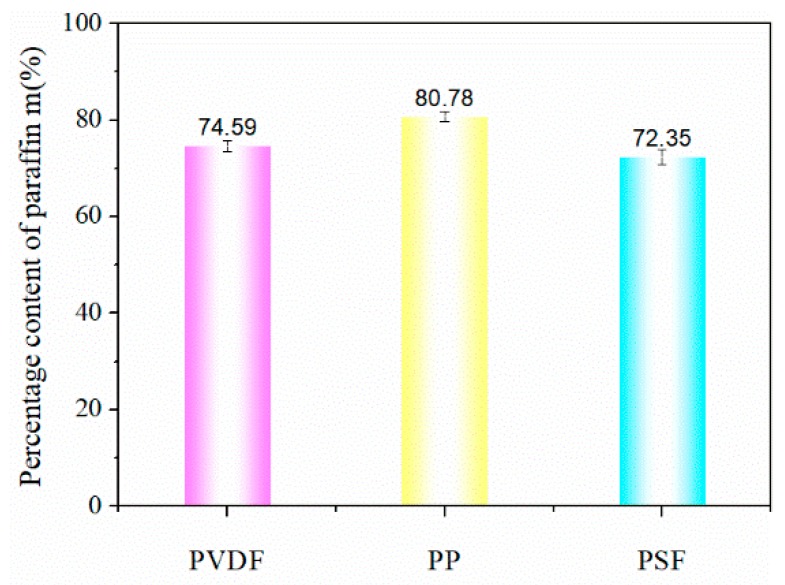
Percentage content of paraffin in the encapsulation membrane.

As per the observed morphology, the three virgin membranes were encapsulated with paraffin but further measurement was required to estimate the actual encapsulation content. In theory, the paraffin encapsulation content of PP hollow fiber membrane should be optimal. The mass before and after paraffin encapsulation was measured by weighing method, and the percentage content of paraffin in the entire encapsulation membrane was calculated. As shown in Figure 5, the paraffin encapsulated in the PP hollow fiber membrane reached 80.78%. As proposed earlier, the porosity is proportional to the amount of paraffin encapsulated.

### 3.5. Chemical Compatibility of Paraffin and Membranes

**Figure 6:**
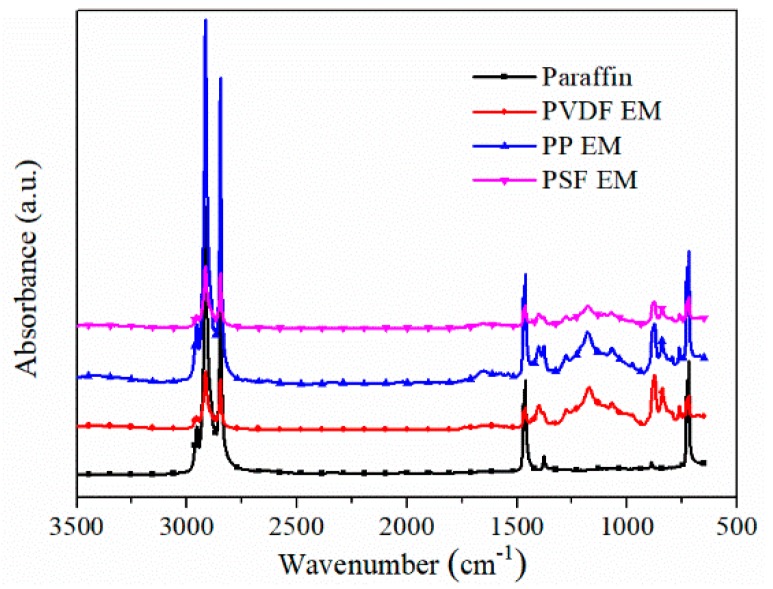
FT-IR spectrum of paraffin and three encapsulation membranes.

Figure 6 exhibits the FT-IR spectra of paraffin and three encapsulation membranes. The characteristic peaks at 2847.50 and 2914.73 cm^−1^ are attributed to stretching vibration of the methylene (CH_2_) in long-chain alkyls [45,46]. The peaks between 750 and 1500 cm^−1^ appear after the introduction of the hollow membrane. The characteristic peak at 1173.34 cm^−1^ is attributed to the C–C stretching vibration of the (CH_3_)_2_CHR [45,46]. In addition, no new peak appeared in the spectrum, revealing that there was physical combination between paraffin and three encapsulation membranes and that no chemical reaction occurred. Chemical reactions would reduce the latent heat of paraffin, and the physical combination was more conducive to maintaining high latent heat of paraffin [47]. In summary, FT-IR results indicate the good chemical compatibility between paraffin and three encapsulation membranes [48].

### 3.6. Latent Heats and Cyclic Reliability of Encapsulation Membranes

**Figure 7:**
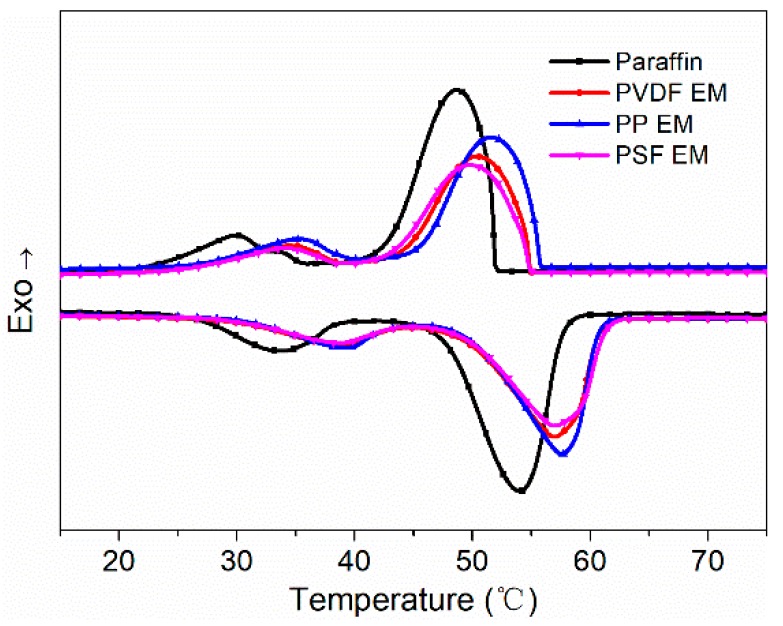
DSC curve of pure paraffin and three encapsulation membranes.

After confirming the successful paraffin encapsulation, the thermal properties of the hollow fiber membranes after encapsulation of paraffin were analyzed. Figure 7 shows the endothermic and exothermic curves of paraffin and three different materials. The endothermic and exothermic curves of paraffin exhibited two peaks during melting and solidification, respectively. The first peak (the temperature was below 40 °C) was attributed to the homogeneously nucleated crystal-rotator transition corresponding to the solid–solid phase transition of paraffin. The second peak was ascribed to the solid–liquid phase change of paraffin from the heterogeneously nucleated rotator-liquid transition [49]. The endothermic and exothermic curves of the three encapsulation membranes also exhibited two melting and solidifying phase change peaks, indicating their similar phase change characteristics to paraffin. The latent heat value was obtained by integrating peak linear with TA Universal Analysis software. In the DSC curve of Figure 7, the curve of the lower half is an endothermic curve, and the temperature at which the state of matter changes from a solid state to a liquid state during the endothermic process is called the melting point. As observed from the melting and solidification process, compared with paraffin, the initial crystallization temperature and the initial melting temperature of the three encapsulation membranes were both increased (Rightward deviation). These results show that the addition of the hollow fiber membranes promotes the crystallization of the paraffin as well as increases the melting point of the paraffin. The underlying cause of this result may be that the melting point of the virgin membrane was higher. After they were combined, the melting point of the composite moved toward the virgin membrane. By comparison of peak strength and peak width of the three encapsulation membranes, we can conclude that the latent heat of PP encapsulation membranes (PP EM) was relatively higher than those of the other two encapsulation membranes. These results are closely related to the encapsulation amount and porosity.

The melting–solidifying properties for paraffin and three encapsulation membranes are displayed in Table 2. The paraffin absorption capacity of the hollow fiber membranes was used to assess the latent heats of the investigated three encapsulation membranes. The theoretical latent heats of the three encapsulation membranes can be estimated by Equation (4), and the results are provided in Table 2 [47,50],

(4) $$H_{C}=\mu \times \Delta H_{p\mathrm{araffin}}$$

where *H*_C_ is the theoretically calculated latent heats, *μ* is the % content of paraffin in the three encapsulation membranes, and Δ*H*_Paraffin_ is the measured latent heat of paraffin in the melting/solidifying process.

The peak temperatures of paraffin during the melting and solidifying process were 54.10 and 48.70 °C, respectively, while the latent heats in the melting and solidifying processes were 138.20 and 145.60 J/g, respectively. In addition, the measured latent heats of the three encapsulation membranes were slightly lower than their theoretically calculated values. After comparing the three encapsulation membranes, we found that the latent heat of PP EM was outstanding whether it was theoretically calculated or actually measured latent heat.

**Figure 8:**
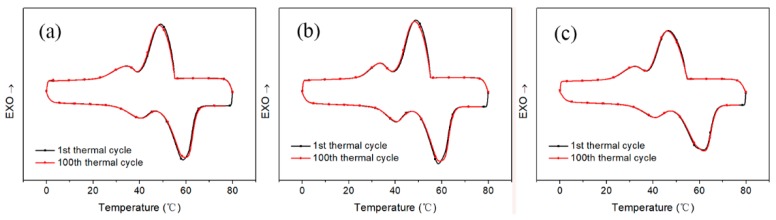
Cyclic reliability of three encapsulation membranes; (**a**) is PVDF EM; (**b**) is PP EM; (**c**) is PSF EM.

For the purpose of exploring the cycle reliability of these encapsulation membranes, these encapsulation membranes were repeatedly melted and solidified 100 times. Since the number of cycles is 100, if the 100 curves were displayed in the same picture, the curves would overlap in a chaotic manner. So only the 1st and 100th data are recorded for comparison. The results of the comparison were shown in Figure 8. Curve (a) has a similar shape to the curve (b), but with careful comparison, the peak of the curve (b) is slightly higher. The specific latent heats retention rate data were shown in Table 3. From the comparison of data of the 1st thermal cycle and 100th thermal cycle, the latent heat retention rate of three encapsulation membranes exceeded 98.5%. This means that the cycle reliability of the three materials are excellent during 100 cycles of repeated melting and solidification.

### 3.7. Thermal Stability and Activation Energy of Encapsulation Membranes

**Figure 9:**
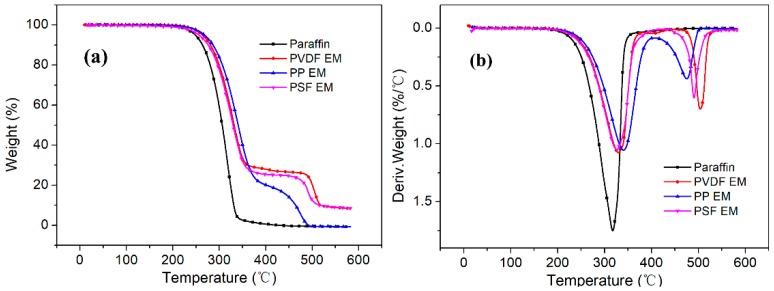
TGA curves (**a)** and DTG curves (**b)** of paraffin and three encapsulation membranes.

Thermo-gravimetric analysis (TGA) is one of the most effective means of evaluating the thermal degradation performance and thermal stability of materials. The thermal stability of paraffin and three encapsulation membranes was studied via TGA and DTG (Figure 9). The thermal decomposition of paraffin displayed a one-step mass loss with the initial thermal decomposition temperature (*T*_−5%_) and the temperature of maximum weight loss rate (*T*_max_) at 248.27 and 317.27 °C, respectively. However, the thermal decomposition processes of the three encapsulation membranes included two decomposition steps. The first degradation step of the three encapsulation membranes started from paraffin degradation. The second degradation step started in the temperature range of 432.37 and 492.54 °C. 

As shown in Figure 9, compared to paraffin, whether it is a TGA or a DTG curve, the initial thermal decomposition temperature (*T*_−5%_) and the temperature (*T*_max_) of maximum weight loss rate of the three encapsulation membranes were shifted to the right. These results indicate that the thermal stability of the encapsulation membranes was improved. However, two peaks appeared in the (b) curve; the first appeared around 350 °C while the second appeared around 500 °C. In the second peak, the temperature (*T*_max_) of the maximum weight loss rate of the PP encapsulation membrane was the lowest, which may be because after the first step, the paraffin was completely decomposed, leaving a part of the pure membrane material. The shift in the second peak is related to the physicochemical properties of the different membrane materials. 

The TGA results also showed that the thermal stability of paraffin could be slightly enhanced via encapsulation in the hollow fiber membranes. The thermal degradation data is presented in Table 4. For PP encapsulation membrane (PP EM), the degradation of the first step started at 268.06 °C, higher than both 263.87 °C of PVDF EM and 256.39 °C of PSF EM. In addition, the temperature (*T*_max_) of the maximum weight loss rate was 240.10 °C, which is also the highest of the three encapsulation membranes. The temperature of the second degradation step had no practical significance and has not been discussed. The PP EM still performed the best among the three studied encapsulation membranes. Whether it is the initial thermal decomposition temperature (*T*_−5%_) or the temperature of the maximum weight loss rate (*T*_max_), PP EM was the best of the three investigated membranes.

To further compare the heat consumed during the decomposition of the three encapsulation membranes, the activation energies of the three encapsulation membranes was calculated using the Flynn-Wall-Ozawa method [42]. These results were employed to analyze the Thermal Decomposition Kinetics. The linear correlation coefficient of the data obtained by this method was close to 1. This result makes the Flynn-Wall-Ozawa method has a good linear correlation at different heating rates and the same weight loss rate. The activation energy *E* was calculated using Equation (3).

**Figure 10:**
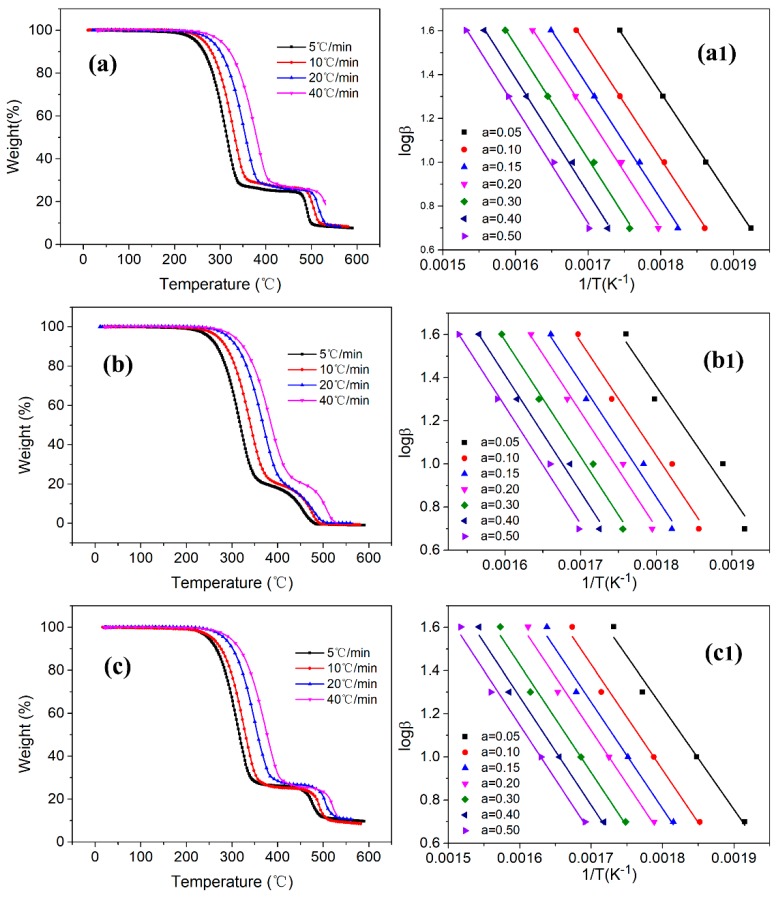
TGA curve of three encapsulation membranes and a linear correlation diagram of Ozawa; (**a**) is PVDF EM; (**b**) is PP EM; (**c**) is PSF EM; (**a**), (**b**), (**c**) are TGA curves with different heating rates; (**a_1_**), (**b_1_**), (**c_1_**) are linear correlation diagrams.

Figure 10 shows the TGA curve of the three encapsulation membranes and a linear correlation diagram of Ozawa while Table 5 shows the calculated activation energy data. As per Figure 10 and Table 5 data, we can conclude that the activation energy of the three encapsulation membranes gradually increased as the weight loss rate increased. When the weight loss rate was 50%, the activation energy of the three encapsulation membranes reached a maximum value, and the three maximum values were 96.02 kJ/mol (PVDF EM), 99.30 kJ/mol (PP EM), and 91.67 kJ/mol (PSF EM). The PP EM consumed the most heat during the decomposition process between 20 to 600 °C. In other words, PP EM stored most energy among the three encapsulation membranes. These results are also consistent with the results of the paraffin encapsulation content. Hence, the larger was the paraffin encapsulation content was, the more was the storage of energy.

## 4. Conclusions

Three phase change energy storage materials with different support layers were successfully prepared and their various properties were systematically compared. The PP hollow fiber membrane was the best amongst the studied membranes as the porosity of the membrane wall was the largest, reaching 85.09%, which led to the highest paraffin content (80.78%) of encapsulation. The SEM characterization results showed that the paraffin was successfully encapsulated in the membrane wall and the columnar hollow part of the membranes.

The latent heat values of the three encapsulation membranes were measured using DSC. The latent heats of PP EM in the melting and solidifying processes were 109.80 and 113.90 J/g, respectively. The latent heat of PP EM was relatively higher than those of the other two encapsulation membranes. 

After the composite phase change materials were prepared, both the initial decomposition temperature and the maximum thermal weight loss were improved, which indicates that the introduction of the membrane as an encapsulation carrier delayed the decomposition of the composite phase change energy storage materials. For PP EM, the initial thermal decomposition temperature (*T*_−5%_) of the first step started at 268.06 °C, higher than both 263.87 °C of PVDF EM and 256.39 °C of PSF EM. In addition, the temperature (*T*_max_) of maximum weight loss rate was 240.10 °C, which was also the highest among those for the three encapsulation membranes. The activation energy during decomposition of the phase change energy storage materials was calculated via the decomposition kinetics. When the weight loss rate was 50%, the activation energy of PP encapsulation membrane reached the highest value at 99.30 kJ/mol. By comparing various properties, PP EM performed the best amongst the studied three encapsulation membranes.

## Figures and Tables

**Table 1:** Materials and equipment used in the experiment.

Category	Model	Use Condition or Characteristic	Suppliers
Twin-screw extruder	SHJ-20	Length-diameter ratios: 40	Nanjing Jieya Extrusion Equipment Co., Ltd., Nanjing, China.
Weight-scale	AE4202	Accuracy: 0.01 g	Shanghai Sunny Hengping Co., Ltd., Shanghai, China.
Vacuum drying oven	DZF6050	Accuracy: 0.1 °C	Shaoxing Supo Instrument Co., Ltd., Shaoxing, China.
Ultrasonic equipment	JP-360ST	Frequency: 28–40 KHZ Heating temperature: 0–200 °C	Shenzhen Jiemeng Cleaning Equipment Co., Ltd., Shenzhen, China.

**Table 2:** Melting–solidifying properties for paraffin and three encapsulation membranes; *H*_M_ and *H*_S_ are the measured latent heats while *H*_C1_ and *H*_C2_ are the theoretically calculated latent heats.

Samples	Melting Process			Solidifying Process		
	*T*_M_ (°C)	*H*_M_ (J/g)	*H*_C1_ (J/g)	*T*_S_ (°C)	*H*_S_ (J/g)	*H*_C2_ (J/g)
Paraffin	54.10 ± 0.27	138.20 ± 2.12	138.20	48.70 ± 0.20	145.60 ± 2.43	145.60
PVDF EM	57.03 ± 0.31	102.20 ± 1.51	103.08	50.52 ± 0.31	107.20 ± 1.39	108.60
PP EM	57.66 ± 0.33	109.80 ± 1.87	111.64	51.67 ± 0.54	113.90 ± 2.17	117.62
PSF EM	57.02 ± 0.29	97.30 ± 1.38	99.98	49.91 ± 0.37	104.40 ± 1.73	105.34

**Table 3:** Latent heat retention rate (*R*_L_) of three encapsulation membranes; *H*1 is latent heat of 1st thermal cycle; *H*100 is latent heat of 100th thermal cycle; *R*L is latent heat retention rate.

Samples	Melting Process			Solidifying Process		
	*H*_1_ (J/g)	*H*_100_ (J/g)	*R*_L1_ (%)	*H*_1_ (J/g)	*H*_100_ (J/g)	*R*_L2_ (%)
PVDF EM	103.53	102.19	98.71	106.37	105.57	99.24
PP EM	108.07	106.95	98.96	111.61	110.86	99.33
PSF EM	99.29	98.34	99.04	102.42	101.17	98.78

**Table 4:** Summary of the thermal degradation data.

Samples	The First Step		The Second Step		Weight Loss (%)
	*T*_−5%_ (°C)	*T*_max_ (°C)	*T*_−5%_ (°C)	*T*_max_ (°C)	
Paraffin	248.27	317.27	--	--	99.98
PVDF EM	263.87	329.52	492.54	503.79	91.64
PP EM	268.06	340.10	450.65	475.41	99.98
PSF EM	256.39	325.84	479.22	490.52	91.46

**Table 5:** Activation energies of samples according to Flynn-Wall-Ozawa’s method.

Conversion *α* (%)	Activation Energy E (kJ/mol)		
	PVDF EM	PP EM	PSF EM
5	91.22	93.56	86.23
10	92.56	95.90	88.93
15	93.31	97.01	89.82
20	93.84	97.49	90.21
30	94.68	98.19	90.77
40	95.27	98.81	91.29
50	96.02	99.30	91.67
